# Image-Based Somatotype as a Biometric Trait for Non-Collaborative Person Recognition at a Distance and On-The-Move

**DOI:** 10.3390/s20123419

**Published:** 2020-06-17

**Authors:** Antonios Danelakis, Theoharis Theoharis

**Affiliations:** Department of Computer Science, Norwegian University of Science and Technology, 7030 Trondheim, Norway; theotheo@ntnu.no

**Keywords:** somatotype trait, somatotype as biometric, biometrics, full body image, deep learning, inception V3 network, siamese network, fusion scheme

## Abstract

It has recently been shown in Re-Identification (Re-ID) work that full-body images of people reveal their somatotype, even after change in apparel. A significant advantage of this biometric trait is that it can easily be captured, even at a distance, as a full-body image of a person, taken by a standard 2D camera. In this work, full-body image-based somatotype is investigated as a novel soft biometric feature for person recognition at a distance and on-the-move. The two common scenarios of (i) identification and (ii) verification are both studied and evaluated. To this end, two different deep networks have been recruited, one for the identification and one for the verification scenario. Experiments have been conducted on popular, publicly available datasets and the results indicate that somatotype can indeed be a valuable biometric trait for identity recognition at a distance and on-the-move (and hence also suitable for non-collaborative individuals) due to the ease of obtaining the required images. This soft biometric trait can be especially useful under a wider biometric fusion scheme.

## 1. Introduction

Biometric recognition at a distance under high security requirements (e.g., border control posts) remains a challenging task and becomes even more so nowadays that “on the move” scenarios are becoming a trend (https://www.schengenvisainfo.com/news/frontex-testing-biometrics-on-the-move-border-check-technology-at-lisbon-airport/). Biometric recognition accuracy largely depends on a set of (complementary with respect to the failure cases) biometric traits under a fusion scheme [1]. It is therefore crucial to expand the set of available biometric traits, especially with traits that can be easily captured by standard sensors, at a distance and on-the-move. One such trait is the somatotype [2]. The somatotype focuses on the measurement of structural aspects of the human body and recent Re-Identification (Re-ID) works show that it can be captured from still full-body images [3].

This paper proposes the use of the human somatotype as a new soft biometric trait [4] based on full-body still images. Current works of the state-of-the-art exploit the somatotype feature for resolving the Re-ID problem which is different to the biometric approach; the Re-ID procedure uses a gallery which is not predefined, meaning that no enrollment has taken place. In this sense, the Re-ID cannot be considered as a biometric problem [5]. Nonetheless, it is possible to compare against Re-ID methods by assuming a fixed gallery and using common biometric evaluation measures; this is the approach taken here and we show that the proposed methods outperform the state-of-the-art in terms of verification, while remaining quite comparable in the identification case.

The somatotype is studied under two common biometric recognition scenarios: (i) identification, and (ii) verification. For the identification case, we implement an appropriately modified variation of the Inception V3 network, while for the verification case, a Siamese network variation is proposed. The resultant deep networks have been trained and assessed on four popular publicly available benchmark datasets: (i) CUHK03 [6], (ii) RAiD [7], (iii) Market-1501 [8] and (iv) RGBD-ID [9,10]. In addition, a much larger dataset has been created by unifying the aforementioned ones and has been used for testing purposes. Finally, an additional synthetic dataset (SOMAset [3]) which is appropriate for testing under different camera angles and cloth variations, is recruited.

### Contributions

The main contributions of the present work are:

1. The proposal of the full-body image-based somatotype as a biometric trait suitable for non-collaborative person recognition at a distance and on-the-move, as well as suitable experiments to verify this.
2. The implementation of two fine-tuned pre-trained deep learning networks (Inception V3 network and Siamese network) for person recognition based on somatotype, trained on publicly available benchmark somatotype datasets.
3. The provision of a somatotype recognition benchmark for this and similar techniques. This benchmark includes a unified somatotype dataset, and the definition of the validation and training sets.
4. Detailed evaluation of the proposed schemes, even for challenging scenarios including camera angle and cloth variations. These evaluations show that the proposed biometric recognition methods based on the somatotype generally outperform that state-of-the-art (which consists of Re-ID methods).

The rest of the paper is organized as follows: After reviewing relevant previous work (Section 2), we describe the implemented methods (Section 3). In Section 4, the experimentation process is presented and corresponding results are illustrated. Section 5 records important observations extracted from the experimental procedure. Section 6 highlights interesting future challenges while Section 7 concludes the paper.

## 2. Related Work

Re-identification work (Re-ID), based on full-body still images, implicitly uses the somatotype feature, especially when training is involved. On the other hand, to the extend of our knowledge, somatotype has been hardly used as a biometric trait for person recognition. Thus, the related work presented in this section, is focused on full-body image-based Re-ID techniques, the closest relative and it shall be used to be compared against our work. Most Re-ID techniques are based on deep learning with Convolutional Neural Networks (CNNs), using the full body images as input and the recognised identity as output, being the most popular [11,12,13]. A number of more complex CNN variations, especially focused on the Re-ID problem, were also introduced; In [14], a duplicate CaffeNet is used. The work in [15], implements a more complex convolutional scheme OSnet, in terms of number of convolutions and kernel sizes, than typical CNNs. Finally, in [16], an extra layer (Eigenlayer) is added to the typical CNN architecture for improving the feature representation. In the aforementioned works, there is usually a pre-processing step which resizes the initial images to the appropriate size expected by the corresponding network. There are also some cases, where data augmentation takes place in order to increase the training samples.

The main drawback with CNNs is that, although they can be well trained and can learn global features, they may lose important local features. Combining learning from global and local features is critical in order to overcome issues related to pose changes, occlusions and non-rigid deformations of the human body. To this end, several models have been proposed such as the Batch DropBlock Network (BDB) [17], the part-based gradation regularization model (PGR) [18], the Multi-Scale ContextAware Network (MSCAN) [19], the Multi Task Deep learning network (MTDnet) [20], the Attribute-Aware Attention Model ($A^{3}M$) [21], the AlignedReID method [22], the pyramidal framework [23], the Integration CNN (ICNN) [24] and the joint attention person Re-ID (JA-ReID) architecture [25].

Furthermore, there are some more methodologies which follow different directions towards solving the Re-ID problem. In [26], the so-called Part-based Convolutional Baseline (PCB) scheme for learning part-level features of the images, instead of global ones, is presented. In [27] a re-ranking process is implemented on the initially retrieved images for leveraging the final result. The methodology in [28] proposes a scheme which resolves the so-called Small Sample Size problem (SSS) [29] (i.e., the training data sample size is much smaller than the feature sample size) which is a common issue that typical distance metric learning techniques have to address. [30] proposes a spatio-temporal person Re-ID (st-ReID) framework that takes into account not only the visual semantic information but the spatio-temporal information as well using consecutive images. A new feature (Local Maximal Occurrence) and a metric learning method (Cross-view Quadratic Discriminant Analysis) is proposed in [31]. In [3], a synthetic dataset (SOMAset) has been used to train an Inception V3-based network (SOMAnet) in order to model additional discriminative information than just the outfit. [32] studies small-sized randomly initialized models. [33] proposes the adaptation of a limited supervised multi-camera Re-ID setup, thus improving the hardware of the entire process. The method of [34] proposes the Auto-ReID framework which enables the automated finding of an efficient and effective CNN architecture for Re-ID. [35] implements a model which involves a generative module for encoding a person’s appearance and structure based on full-body images, and a discriminative module that shares the appearance encoder with the generative module. Finally, the work of [36] collects and evaluates training lessons from the literature to improve performance.

Finally, it should be highlighted that there are very interesting state-of-the-art works which either perform Re-ID based on different modalities than full-body still images derived by other sensors than conventional cameras [37,38,39], or combine the somatotype trait with other biometric modalities (i.e., face) in order to perform improved biometric recognition [40]. Nonetheless, the scope of this paper is focusing on full-body still images derived by standard 2D cameras.

## 3. Methods

After discussing the somatotype as a biometric trait, we present its use in both identification and verification scenarios along with proposed deep learning based schemes.

### 3.1. Somatotype as a Biometric Trait

Somatotype focuses on the measurement of structural aspects of the human body, and includes three main somatotype categories [41]: (i) Ectomorph: long and lean body figures, with little body fat and muscle; (ii) Mesomorph: athletic, solid and strong body figures; (iii) Endomorph: body figures with lots of body fat. Although the human somatotype has (implicitly) achieved remarkable results in the Re-ID field (by using full-body images of humans), as presented in Section 2, it has never before been used in recognition scenarios, to the best of our knowledge. The Re-ID procedure uses a not pre-defined gallery (no enrollment takes place), thus it cannot be considered as a biometric problem [5]. It was not until the work of Barbosa [3] that somatotype was explicitly recognized as a useful trait for the Re-ID problem. In [3], synthetic data with varying somatotypes were used, to show that this trait is capable of re-identifying human beings even after a change in apparel, based on learning somatotypes. It thus makes sense, as a continuation of the aforementioned work, to explore the full-body image-based somatotype trait in non-collaborative person recognition at a distance and on-the-move, potentially opening up a new biometric field.

Since the somatotype is based on a simple whole body image, it can be captured using off-the-shelf cameras, from a distance and on the move. Such cameras are already in place in areas where security is controlled (e.g., airports, ports, malls). Somatotype is also available in databases of law-enforcement agencies which typically include a full-body image of subjects.

### 3.2. Identification Scenario

This scenario studies the usability of the somatotype trait for person identification. A gallery dataset of identities, containing one or more somatotype instances for each identity is assumed. This gallery dataset would normally be the result of an enrollment process and is used for training. Unknown query somatotype instances are then presented and the trained system is expected to map these queries onto identities of the gallery.

#### 3.2.1. Inception V3 Network Architecture for Identification

The Inception V3 network [42], which has proven extremely successful in classification problems (See *ReCeption* method in classification part of Task 2a of ILSVRC 2015 rankings: http://image-net.org/challenges/LSVRC/2015/results), has been implemented for the identification scenario. This is an improved version of a series of deep networks starting with Inception V1 network, widely known as GoogleNet [43]. GoogleNet recruits convolution filters with multiple sizes which operate on the same level. This is done in order to learn salient parts of the input that can be of various sizes. Inception V2 [42] factorizes a 5 × 5 convolution into two 3 × 3 convolution operations to improve computational speed and reduce the representational bottleneck.

**Figure 1:**
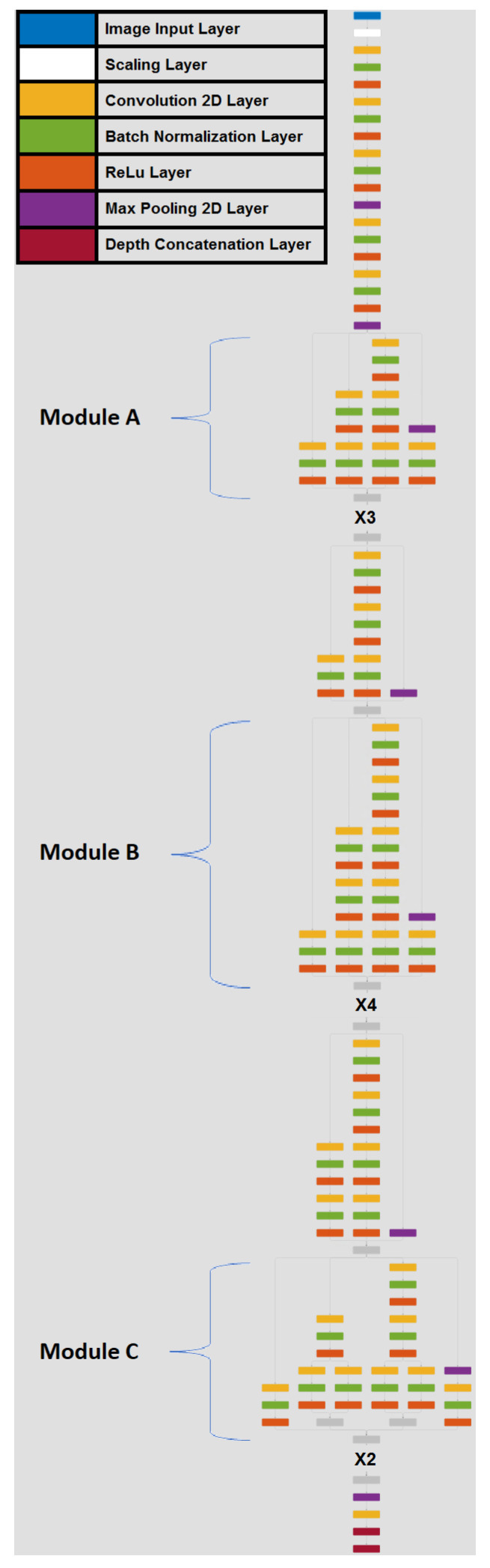
Inception V3 network architecture as implemented in the current work.

Finally, Inception V3 has adopted some upgrades regarding the convolution filter size, the auxiliary classifiers and the prevention of over-fitting. Furthermore, this version of Inception is capable of encoding more attributes related to the identity of a person (i.e., height, obesity and gender) in addition to outfit and body physique [3]. This is the main reason that the aforementioned deep network was chosen. Figure 1 illustrates the Inception V3 network architecture as implemented in this work. There are three different types of modules in the Inception V3 network: (i) Inception *A*, (ii) Inception *B* and (iii) Inception *C* module. All modules are appropriately designed for generating discriminatory features and for reducing the number of trainable parameters. Each module is composed of several convolutional, batch normalization, ReLU and pooling layers. Modules *A* and *C* use small convolutional layers which are responsible for the reduction of the trainable parameters of the network. On the other hand, module *B* uses mostly large convolutional layers for feature extraction. As illustrated in Figure 1 three Inception *A* modules, four Inception *B* modules and 2 Inception *C* modules are stacked sequentially (indicated by notation “X3”, “X4” and “X2” in the figure respectively).

#### 3.2.2. Identification Procedure

For each training sample *x*, the Inception V3 network computes the probability of it belonging to each class k∈1…K as indicated in Equation (1). Cross entropy is defined as the loss function of the network (see Equation (2)).

(1) $$p\left(k|x\right)=\frac{exp(z_{k})}{\sum_{i=1}^{K}exp\left(z_{i}\right)}$$

(2) $$l=-\sum_{k=1}^{K}log\left(p\left(k\right)\right)\cdot q\left(k\right)$$

where $z_{i}$, p(k) and q(k) correspond to the output of the network, the predicted class distribution and the ground-truth class distribution respectively.

Transfer learning has been implemented on the pre-trained Inception V3 network. To this end, the final two layers of the pre-trained network have been adapted to each dataset used in the experimentation process. The training set of each dataset is considered as the identities gallery database. The number of outputs (i.e., classes) of the final network equals the number of different identities in the gallery. Each query results in a confidence score in every output of the network and the query is mapped to the class with the highest confidence score. Should the highest confidence score does not exceed a specified threshold ϵ, then the identity of the query is considered to be unknown (i.e., no such identity in the gallery).

#### 3.2.3. Verification through Identification

The aforementioned scheme can be adopted for verification. In this case, a post-processing operation is implemented. The identity of an individual is verified if the output of the system for the class of the claimed identity has a confidence score higher than a predefined threshold ϵ.

### 3.3. Verification Scenario

This scenario studies the usability of the somatotype trait for identity verification. A gallery biometric instance (representing the claimed identity) and a query instance (representing the claimant) are compared by a pre-trained system, producing a similarity score. A confidence threshold ϵ on this score decides whether the claimed identity is verified.

#### 3.3.1. Siamese Network Architecture for Verification

For the verification scenario a Siamese network [44] is adopted. Due to the nature of these networks, they are ideal for verification tasks of reduced dimensionality [45]. Siamese networks have mainly been used for face verification, with DeepFace [46] and FaceNet [47] being the most popular implementations. Within the context of the current work, their implementation is extended to somatotype-based verification.

Siamese networks are fed with two somatotype biometric instances and produce a similarity score as output in the interval [0, 1]. A similarity score close to 0 indicates that the identity of the two inputs is different, while close to 1 that it is the same. The response time is extremely fast. As mentioned already, in the case of identity verification, the first biometric instance will be taken from a gallery dataset which is constructed during an enrollment process. The second biometric instance will typically be captured by a biometric sensor (2D camera) on-the-fly while an individual walks through a security area, having declared a claimed identity. A one-to-one comparison takes place; a similarity score over a predefined confidence threshold ϵ confirms the person’s identity.

It should be highlighted that, re-training of the Siamese network for a new gallery dataset is not required (e.g., when more identities are added to the gallery). This is because the One-Shot-Learning [48] procedure is used and happens for two reasons:

1. Unlike the Inception V3 network, whose output is a *k*-dimensional vector (where *k* is the number of identities) and this dimension can be modified as the number of identities changes, the Siamese network outputs a scalar similarity score in the interval [0, 1] for any input pair. This means that the output dimensionality remains constant at all times.
2. The Siamese network is actually trained to learn the similarity function instead of the biometric data itself. This means that once the network is trained on a specific type of data, it knows how to compute the similarity between new biometric instance pairs.

Unlike the typical deep learning networks, Siamese networks are trained to learn how to compare two images rather than the training images themselves. Thus, they only need a small training sample to be trained upon. In theory, this could even work with a training set containing only one instance per identity.

**Figure 2:**
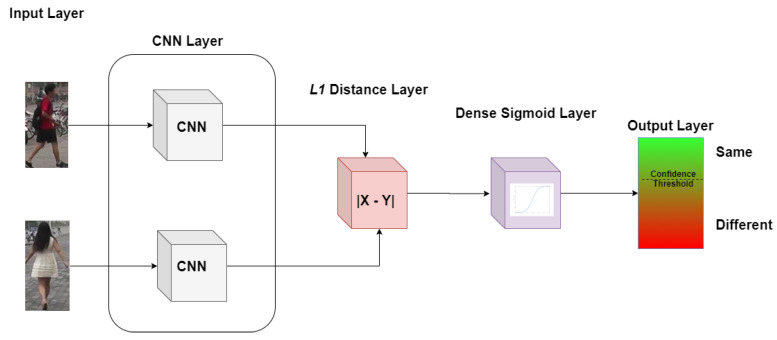
Siamese network architecture as implemented in the current work.

**Figure 3:**
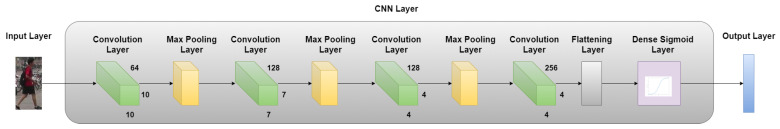
Convolutional Neural Network (CNN) architecture as utilised within the Siamese network of the current work.

Each of the two biometric input instances of the Siamese network are fed into two separate but identical CNNs. Then, the output of the CNNs is merged using the $L_{1}$ distance (see Equation (3), where *l* denotes the output dimension of the CNNs). The estimated $L_{1}$ distance value is passed through a sigmoid function which produces the similarity score as the network’s output. Each CNN begins with three consecutive pairs of convolutional and max pooling layers. The first convolution layer uses 64 filters of dimension 10 × 10. The second convolution layer uses 128 filters of dimension 7 × 7. Finally, the third convolution layer uses 128 filters of dimension 4 × 4. All three layers use ReLU as their activation function. The third max pooling layer is succeeded by another convolutional layer using 256 filters of dimension 4 × 4 and ReLU activation function. A flattening layer follows before the final dense layer with the sigmoid activation function. Figure 2 illustrates the Siamese network architecture as implemented in this work. Figure 3 illustrates the architecture of each CNN utilized within the Siamese network.

(3) $$L_{1}=-\sum_{i=1}^{l}\left|CNN{\left(out\right)}_{1}^{i}-CNN{\left(out\right)}_{2}^{i}\right|$$

#### 3.3.2. Identification through Verification

The aforementioned scheme can be also utilised for identification purposes. In this case, the query will be compared against every single instance of the gallery set. To this end, each biometric pair that is formed by the query and each instance of the gallery set will be consumed by the Siamese network as input. If the highest similarity score achieved exceeds a specified confidence threshold ϵ and the gallery part of the input pair that achieves the higher score is of the same identity as the query part, then the identity of the query is verified by the system.

## 4. Experiments

Experimentation was conducted on four popular, publicly available datasets, one additional unified dataset and one dataset appropriate for testing variations in camera angle and apparel. The current section illustrates the datasets and the corresponding performance results and gives a comparison of the proposed framework against the state-of-the-art methodologies for each scenario. The methodologies that we compared against came from the Re-ID field. Despite the fact that Re-ID and recognition are not the same tasks [49], we chose to compare against such techniques since, to the extent of our knowledge, there were no previous image-based somatotype recognition techniques to compare against and Re-ID was the closest relative. This is also indicated in [50]. Furthermore, the same evaluation metrics could be recruited.

### 4.1. Datasets

A brief description of the datasets that were used for experimentation follows. *CUHK03:* CUHK03 is the first relevant dataset large enough for deep learning [6]. It contained 13,164 images of varying dimensions and consists of 1467 identities. The images were taken from five cameras with different acquisition settings. The dataset version where pedestrians in the raw images were manually cropped was selected in order to keep the process simple. Testing (100 identities) and training (1367 identities) partitions were also provided and used for assessment.

*RAiD:*RAiD [7] is another related dataset where a limited number of identities (41) was captured and contained 6920 images in total. The dimension of each image was 128 × 64. The images were collected using a four camera setting (two indoors and two outdoors). The subjects were randomly divided into two sets, training (21 identities) and testing (20 identities).

*Market-1501:*Market-1501 is the largest relevant real-image dataset [8]. It contained 1501 identities and over 32,668 images. The resolution of the images was 128 × 64. Five high-resolution and one low-resolution camera were recruited for acquisition. The training set consisted of 750 identities while the testing set consisted of 751 identities.

*RGBD-ID:*RGBD-ID [9,10] dataset was captured using RGB-*D* cameras. Although it only included 11 identities, RGBD-ID contains many instances for each identity as well as images of the same identities wearing different clothes, which made this dataset very challenging. The total number of images was 22,845 and the image dimension was 640 × 480. Testing and training images were also provided.

**Figure 4:**
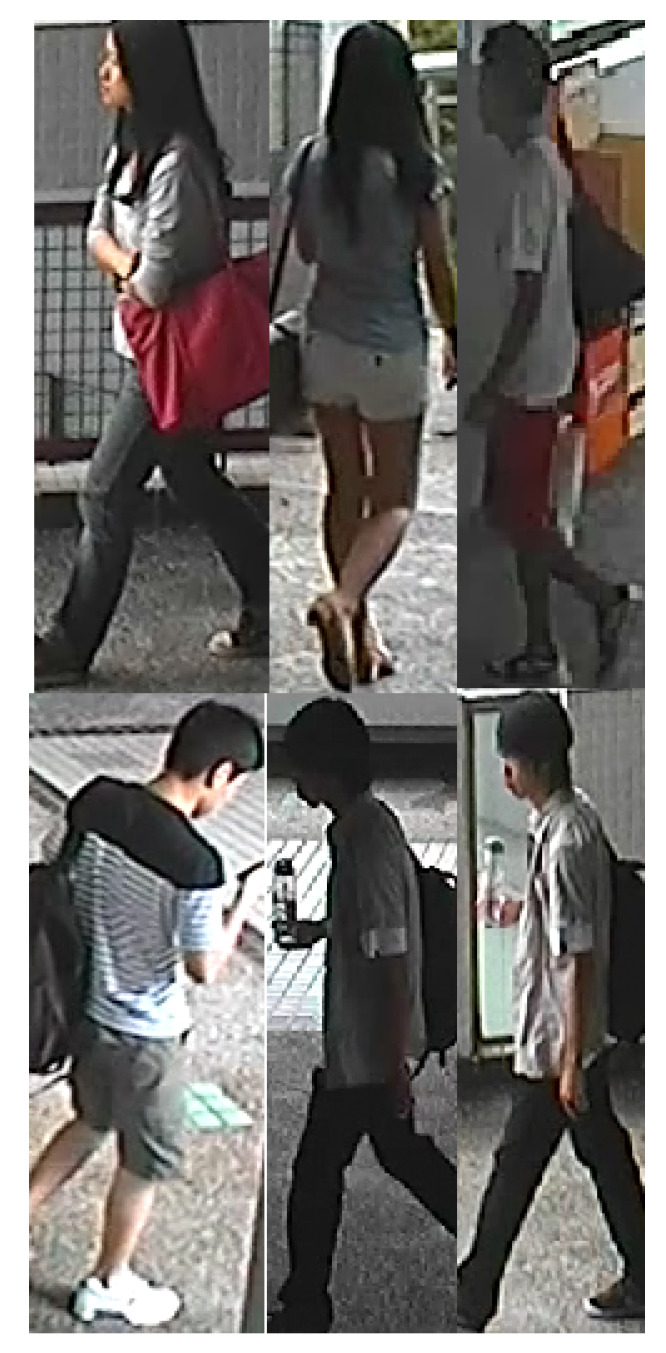
CHUK03 dataset exemplary samples.

**Figure 5:**
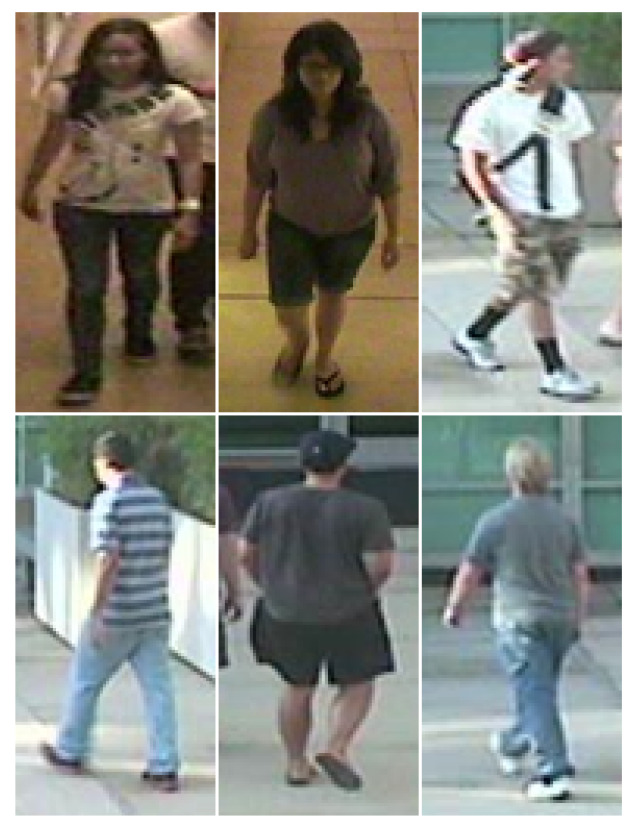
RAiD dataset exemplary samples.

**Figure 6:**
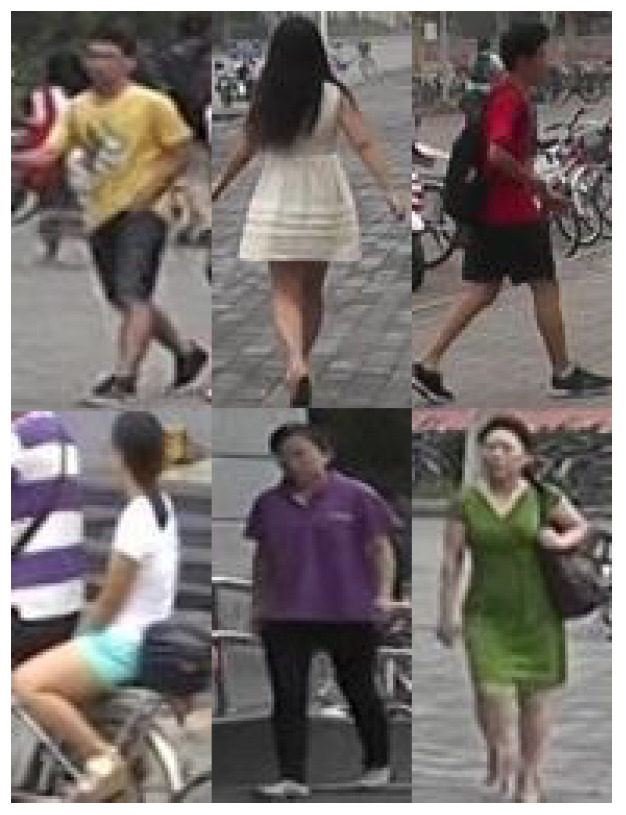
Market-1501 dataset exemplary samples.

**Figure 7:**
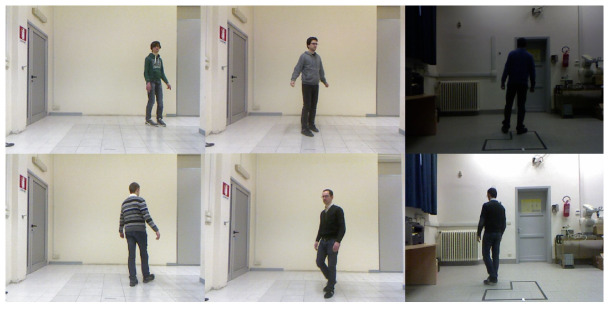
RGBD-ID dataset exemplary samples.

Figure 4, Figure 5, Figure 6 and Figure 7 illustrate some example images from the four datasets.

*Unified dataset:* We created the Unified dataset that combined all the aforementioned publicly available datasets with the aim of maximizing the number of identities, which had a great influence on training and testing, as well as diversifying across different acquisition devices and protocols. In addition, all unique characteristics of each dataset were kept and combined, thus, creating a more challenging dataset. This dataset contained six randomly selected image instances for each identity of the initial datasets. This number was selected two reasons: (i) on the one hand, it excluded just a small number of available identities (identities with less than six recorded instances are skipped but there are only just a few such identities), and (ii) it was a realistic number of images that could be recorded during an enrollment procedure (enrollment in real-world conditions should not be a time-consuming process for the enrolled individual [51]). In total, there were 2939 identities and 17,634 images. The dimension of the images was resized to 299 × 299 which was the standard dimension of the Inception V3 network input. This was done by applying bilinear interpolation (i.e., the output pixel value was a weighted average of pixels in the nearest 2 × 2 neighborhood). Out of the six instances per identity, five were randomly selected as the training set. The remaining instance belonged to the testing set. Furthermore, there was the case where an identity was considered to be unknown. In that case, all six instances of the identity belonged to the testing set. Due to the terms of use of the four initial datasets, the Unified dataset could not be directly re-distributed. Nonetheless, we provided the filenames or indices of the selected images, so that one could easily reproduce the dataset.

**Figure 8:**
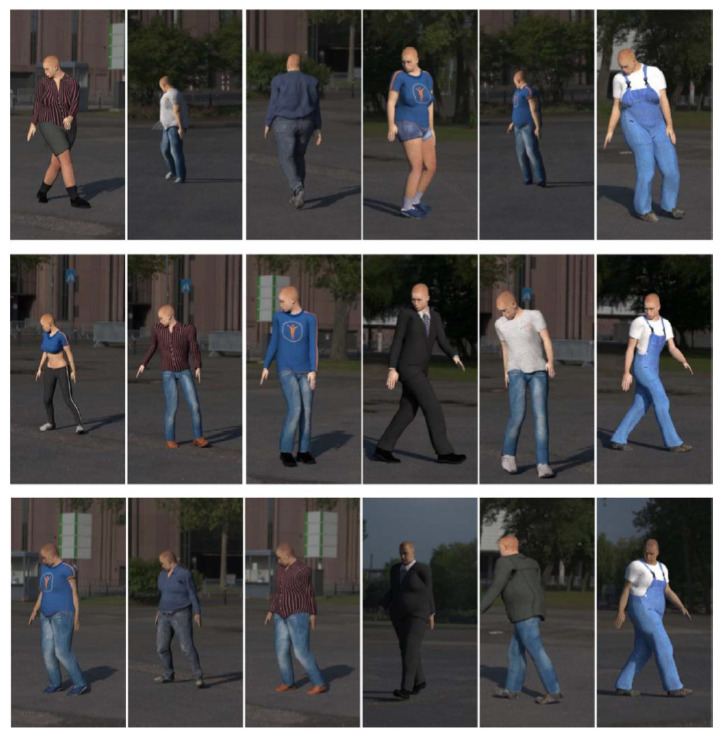
SOMAset dataset example images.

*SOMAset dataset:* Finally, the SOMAset dataset [3] (https://www.kaggle.com/vicolab/somaset) was used in order for the proposed scheme to be tested specifically under different clothing and pose variation scenarios. SOMAset is a synthetic somatotype dataset which includeds 50 identities (25 males and 25 females). It also accounted for different ethnicities. Each identity wore eleven sets of clothes and assumed 250 different poses, over an outdoor background scene, with varying illumination conditions. This made SOMAset ideal for testing the clothing and pose variation effect on the proposed deep learning scheme. Each image in the dataset had dimensions of 200 × 400 pixels. Figure 8 illustrates example images of SOMAset. Table 1 sums up all the recruited datasets along with their main characteristics. It is important to highlight that, although all datasets contained multiple poses for each subject included, only SOMAset provided a standard pattern to facilitate the elaboration, using programming procedures, of both the pose and clothing variation in the defined experimentation protocol. That is why the aforementioned dataset was chosen for the corresponding experiments.

### 4.2. Results

#### 4.2.1. Identification Scenario

The identification module of the Inception V3 network, as described in Section 3, is strictly used experimenting on the identification scenario. The identification experiments were conducted using MATLAB toolboxes on a Windows 10 PC with an Intel i9 CPU at 2.30 GHz, 32.00 GB RAM and a NVIDIA Quadro T2000 GPU of 4 GB memory.

**Figure 9:**
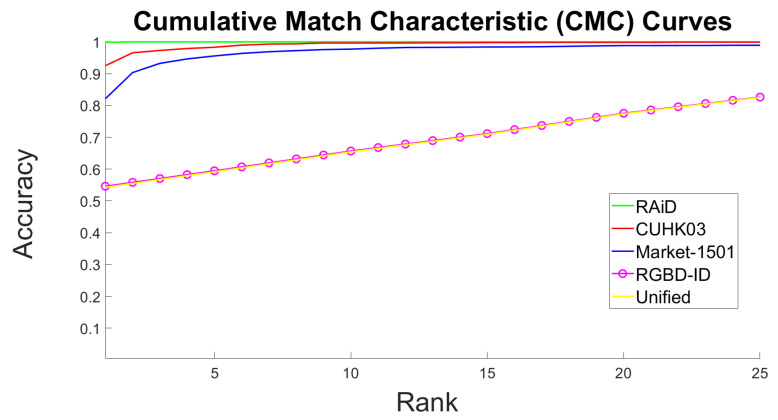
Cumulative Match Characteristic (CMC) curves per dataset as achieved by the Inception V3 network.

Table 2 illustrates the accuracy at rank-1, 5 and 10 of the proposed scheme. Fortunately, it is possible to compare the proposed identification methodology against state-of-art Re-ID methods since rank-*n* rate is a common way of evaluating Re-ID methods; they are essentially considered as retrieval problems. The Cumulative Match Characteristic (CMC) curves of the proposed somatotype identification system for all datasets are illustrated in Figure 9 and confirm the results presented in Table 2. Note that, the best curve was achieved for the RAiD dataset (green color). The curve which corresponds to the RGBD-ID dataset (magenta color) almost overlaps with the one of the Unified dataset (yellow color). Nonetheless, the CMC curve of the Unified dataset was a bit lower which made it the most challenging case to handle. Table 3 compares the accuracy (at most popular ranks of the state-of-the-art, i.e., rank-1, 5 and 10) of the proposed scheme against recent top-ranked state-of-the-art methods for each dataset. ‘N/A’ stands for not available value. Some methodologies achieved better results on one dataset (i.e., Market-1501) but worse on another (i.e., CUHK03) compared to the proposed method. It is worth highlighting that all our results were achieved in a single query setting without using any re-ranking algorithms.

##### Clothing and Pose Variation Effects

Variations in attire and pose are probably some of the most challenging in recognizing somatotype. To this end, experiments have been conducted on the SOMAset dataset, which includes such variations, in order to see how the proposed scheme deals with very challenging data.

**Effect of Clothing Variation** All 250 poses for each identity wearing a specific outfit were chosen as the training set. As testing set, a subset (10%) of the poses for each identity wearing an outfit different to the one corresponding to the same identity in the training dataset, were chosen. Thus, all queries are identities with different outfit to the one used in the training of the system on the same identity. This was significantly harder than the scenario employed in the other state-of-the-art methods using the RGBD-ID dataset where only a subset of the query identities wore different clothes to the ones in training set. The Rank-1 score dived to 15.70%, highlighting the fact that distinguishing identity based on somatotype under different clothes is a major challenge. This is the main reason for the performance decrease of the proposed scheme in the RGBD-ID and the Unified datasets. Table 4 illustrates the rank scores of this experiment.

**Effect of Pose Variation** In the pose variation case, the experiments were split into three sub-cases: (i) frontal pose, (ii) back pose and (iii) side pose. For each sub-case, all images of a specific pose (frontal, back or side) for each identity wearing a specific outfit were chosen as training set. As testing set, a subset (10%) of the remaining poses for each identity wearing the same outfit as in the training dataset, were chosen. Thus, all queries are identities with the same outfit but in a different camera angle that the one used for the training of the system on the respective identity. This was significantly harder than the scenarios of mixed poses employed in the other state-of-the-art methods. Table 5 illustrates the accuracy at different ranks. It appeared that the system was better trained with the side pose images. This is in agreement with the results illustrated in [52]. Thus, pose variations can be addressed especially when appropriate training samples are used.

**Figure 10:**
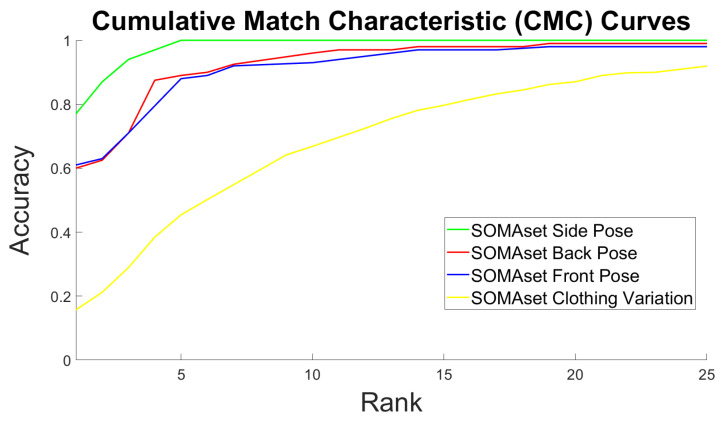
SOMAset dataset CMC curves for different poses and clothing variation as achieved by the Inception V3 network.

The corresponding CMC curves of the aforementioned clothing and pose variation effect experimentation are illustrated in Figure 10. The curves visually confirm the results of Table 4 and Table 5. The best curve was achieved when training the network with the side pose (green color) images. The curves corresponding to training with the back (red color) and front (blue color) images followed, while the most difficult effect to be handled was the clothing variation with the corresponding (yellow color) curve being at the bottom.

#### 4.2.2. Verification Scenario

The verification module of the Siamese network, as described in Section 3, was strictly used experimenting on the identity verification scenario. The verification experiments were conducted using Python on a Windows 10 PC with an Intel i9 CPU at 2.30 GHz, 32.00 GB RAM and a NVIDIA Quadro T2000 GPU of 4 GB memory.

**Figure 11:**
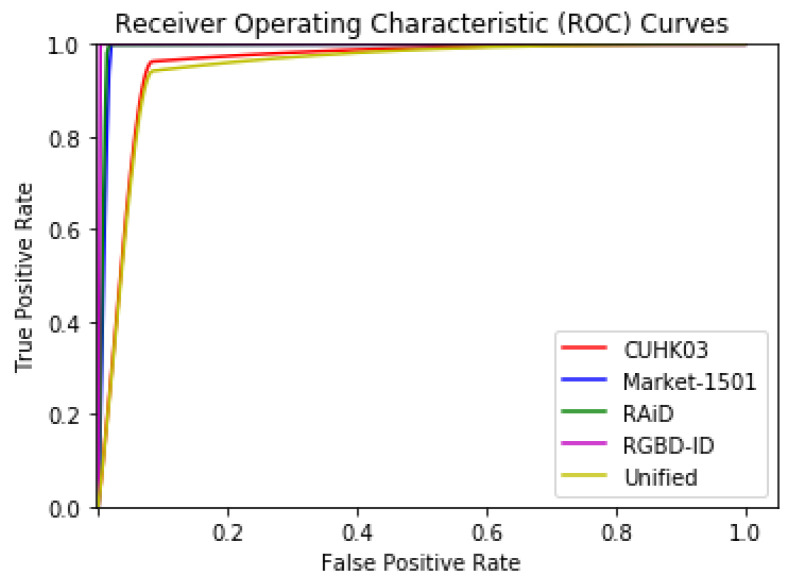
Receiver Operating Characteristic (ROC) curves per dataset as achieved by the Siamese network. Note that the green and blue color lines almost overlap.

Table 6 illustrates the verification accuracy achieved on the evaluation datasets. Note, that this was not the same as identification rank-*n* scores. Figure 11 illustrates the Receiver Operating Characteristic (ROC) curves for the proposed somatotype verification system for all datasets. The ROC curve is commonly used for evaluating verification experiments. The curves confirmed the results of Table 6. The best curve was achieved for the RGBD-ID dataset (magenta color) with the curves of RAiD (green color), Market-1501 (blue color), CUHK03 (red color) and Unified (yellow color) dataset following. The curves corresponding to RAiD and Market-1501 datasets almost overlapped with the one of the RAiD being slightly better. The Unified was, as expected, the most challenging dataset. Table 7 presents the mean Average Precision (mAP) of the proposed verification method compared to recent top-ranked state-of-the-art Re-ID methods, for each dataset. The mAP was used in the evaluation of both identification and verification experiments; a comparison of the two proposed learning methods (Siamese vs. Inception V3) is thus illustrated in Table 8 via their mAP values per dataset.

##### Clothing and Pose Variation Effects

**Figure 12:**
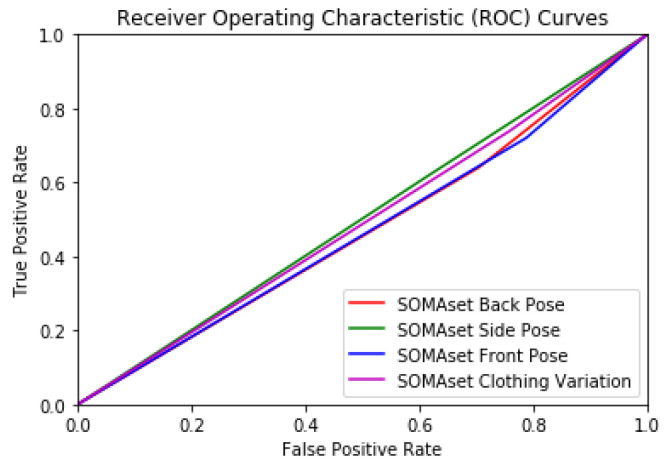
SOMAset dataset ROC curves for different pose and clothing variations as achieved by the Siamese network.

The verification scenario methodology was also tested under clothing and pose variations. Experiments were conducted on the SOMAset dataset. The experimental protocol was the same as in the identification scenario. In contrast to the identification methodology (Inception V3 network), it seemed that the Siamese network was less sensitive to cloth variations but slightly more sensitive to camera pose variations. The verification accuracy is presented in Table 9. The ROC curves are illustrated in Figure 12. Once again, side pose (green color) achieved better results than when front (blue color) and back poses (red color) were used in training. The clothing variation case (magenta color) seemed to be more stable in the Siamese rather than in the Inception V3 network.

### 4.3. Implementation Details

The only undertaken pre-processing step for both identification and verification scenario is that the initial images were resized in order to feed the input of the corresponding network. For the identification experiments, the learning rate of the training process was set to 0.0003, the size of the mini batch to 10 and the training epochs to 12 for the four publicly available datasets and to 30 for the unified dataset. For the verification scenario, the learning rate of the training process was set to 0.00006, the size of the mini batch to 32 and the training epochs to 30 for the unified somatotype dataset. The values of the aforementioned hyper-parameters were chosen experimentally. After training, the testing process took place without an intervening validation phase.

As the confidence threshold ϵ for both scenarios, the value 0.7 was chosen. It should be mentioned here that, the threshold was parameterised in the proposed scheme. Thus, any desired threshold could be chosen. During this research, many different thresholds were tested and the 0.7 threshold value was experimentally proved to achieve the best results.

### 4.4. Timings

The time needed on a per-query basis for both scenarios was very low. More precisely, for identifying a query, the time required by the Inception V3 network was approximately 0.05 s, while for verifying an identity the required time by the Siamese network was approximately 0.03 s.

If the Inception V3 network was implemented for verification, the per-query time was not much different to that for the identification case, as the same process took place followed by an identity confirmation post-processing which was of *O*(1) time complexity. On the contrary, if the Siamese network was implemented for identification, the per-query time drastically increased. That is because it should be multiplied by the number of instances of each identity in the training set of the corresponding dataset. If we consider that there are *m* instances for each identity and *n* identities in total, then the per-query time required will be multiplied by a factor of *O*(*n* × *m*). Thus the bigger the dataset the more the time needed for the system to respond. In our case, this time varies from approximately 3 min for the RAiD dataset to approximately 10 min for the Market−1501 dataset.

## 5. Discussion

Identification and verification are biometric recognition problems with different evaluation metrics. In the case of identification, CMC curves and Rank-*n* accuracy (n=1,5,10) are often used. Respectively, in the case of verification, ROC curves, mAP and VerificationRate are commonly used. The nearest comparable state-of-the-art methods that have implicitly or explicitly (only [3]) used the somatotype feature have focused on Re-ID, which is an identification problem. Thus, although the proposed identification method could be directly compared against these state-of-the-art Re-ID methods, this was not the case for the proposed verification method. To overcome this problem, mAP values were used, as mAP is common to both problems. The only drawback is that mAP is not made available as often as other measures in related publications.

The experimental results for the identification scenario were very promising for the majority of the cases. However, in more challenging datasets which include a change of attire (RGBD-ID and Unified), identification accuracy drops significantly. Thus, while this method may be suitable for constrained cases where individuals are not expected to change attire (e.g., intra-day), it does not appear suitable for identification across longer time periods, where a change of attire is expected. Another problem is that it necessitates time-consuming retraining for every addition to the gallery dataset, making it unsuitable for dynamic galleries of identities. Still, given the ease of capturing the somatotype (still images) it can be an extremely useful and cheap component under a biometric fusion scheme. At the recognition phase, no collaboration is required, since somatotype is based on simple still images of a person’s body; the person may or may not have enrolled.

The results of the verification scenario indicate that the proposed method achieves very high verification rates for all datasets. Furthermore, the scheme surpasses state-of-the-art performance, including the Inception V3 network approach, for every single dataset used for experimentation. The increased verification rates in both RGBD-ID and Unified datasets indicate that, unlike the Inception V3 network, this scheme is much less sensitive to cloth variations. On the other hand, this scheme is slightly more sensitive to pose variations. That can be explained by the fact that the Siamese networks are trained on a comparison metric rather than the images themselves. Thus, if it is trained only in comparing over a specific pose, then comparing over a different pose will be an unknown for the system. An important advantage of the Siamese network is that it needs no re-training when utilised with different datasets as it follows the One-Shot-Learning approach. One can thus assume a dynamic gallery dataset without the time cost of re-training. Due to the nature of the Siamese networks, even the initial training set doesn’t have to be as extensive as for typical deep networks. However, when aimed at the identification scenario, the Siamese network is more time-consuming than the Inception V3 network, as its performance is linear in the size of the gallery rather than constant.

The effect of clothing is a major issue that has been highlighted in this paper. Ideally, a person recognition system should be invariant to clothing, as clothing is not a characteristic of a person’s identity. However, since the input modality is images, such a recognition system cannot be completely invariant to clothing or for that reason other image artifacts. Just as a facial recognition system cannot be completely invariant to occlusions. However, here comes the advantage of being able to train on a relatively large dataset with exact ground truth; the machine learning system learns the underlying somatotype because it is presented with the same somatotype wearing different clothes as well as different somatotypes wearing the same clothes. Still, there is the question of what the network actually learned. It was thus deemed useful to investigate whether the network, after training, is better at distinguishing somatotypes or clothes. Note that the latter would seem like an easier task, as it is based on the outer appearance that is obvious in full body images. The mean value and standard deviation of the similarity score for the same subjects wearing different clothes as well as different subjects wearing the same clothes are shown in Table 10 for a balanced set of five different cloth variations and five different somatotype variations. The number of total cloth variations that are common for males and females is five, and that is why this number is chosen. Of course, this number is smaller than the number of somatotype variations in the original dataset. This led to the use of 6250 somatotype images, which is less than the 10% of the full dataset. The mean values corresponding to subjects of the same somatotype are slightly higher than the ones corresponding to subjects wearing the same clothes, while the standard deviations corresponding to subjects of the same somatotype are negligible, in contrast to the ones corresponding to subjects wearing same clothes.

An exhaustive study regarding the influence of the different movements of the structural aspects of the human body on the derived accuracy is not applicable for the time being. Due to the plethora of such movements this would require many resources and would be very time-consuming. Moreover, the very nature of the datasets themselves make the aforementioned study unrealistic. The datasets contain only still images which cannot capture the entire movement spectrum of the structural aspects of the human body. In that sense, the assessment of the proposed deep networks under three different pose variations can be thought of as an assessment under a small subset of different movements of the structural aspects of the human body. As mentioned in Section 6, the recruitment of videos (image sequences) instead of the still images, adding extended temporal information as well, can offer a better basis for such a study, but the problems of resource allocation and time complexity will remain, possibly restricting the study just to a bigger movement subset than the one analysed here.

Summing up the discussion, Table 11 illustrates the advantages and disadvantages of the two proposed schemes.

## 6. Future Challenges

One promising way of extracting more accurate somatotype features and thus potentially improving accuracy when there exist variations in attire, is the use of video sequences instead of still images. Thus, the entire movement spectrum of the structural aspects of the human body could be encoded by the deep networks. Preliminary works in this direction could be used as a basis [55,56]. Moreover, synthetic somatotype datasets (e.g., SyRI [57], SOMAset [3] and PersonX [52]) are extremely useful in training, especially for creating controlled subject variations in e.g., attire and pose. In the future we expect such datasets to be of increasing importance in transfer learning scenarios, combined with emerging more sophisticated deep networks such as Inception V4 [58] and ResNet [59].

## 7. Conclusions

The human somatotype, as can be captured from simple full-body images, is an extremely attractive biometric trait as it can be used on-the-move and at a distance, thus being suitable even for non-collaborative subjects. In addition it requires no specialised capture equipment. This paper studied the possibility of using the somatotype as a biometric recognition trait and proposed two respective learning networks for both identification and verification scenarios. Evaluation has been conducted on well-established publicly available datasets for Re-ID, as well as on a new unified dataset that was created by merging the aforementioned ones and contains the largest number of identities.

The proposed Inception V3 network is suitable for non-collaborative and on-the-move identification and/or verification using a pre-constructed, static, gallery dataset of identities. However, application of the scheme on a dataset of identities that can be dynamically modified is problematic, because the Inception V3 network should undergo a time-consuming re-training process for every change in the gallery. Moreover, this scheme is sensitive to cloth variations. On the other hand, the proposed Siamese network scheme is suitable for non-collaborative identity verification on-the-move. It can also be utilised for the identification process at the cost of a linear time penalty. The implementation of One-Shot-Learning ensures that system training is performed only once and offline, making it suitable for a dynamically varying gallery.

Under constrained conditions, where individuals are not expected to change attire across captures and where recognition failures are not catastrophic (e.g., access to non-critical events), the somatotype feature appears extremely attractive due to the ease and low-cost associated with its capture. However, under conditions of large time ranges, where individuals are expected to change attire across captures, the somatotype trait may not be adequate as a stand-alone biometric, particularly if high recognition accuracy is required. Still, under such more general conditions, the somatotype is useful under a biometric fusion scheme, where it can boost a biometric system’s performance at a very low cost.

## Figures and Tables

**Table 1:** Sum up of the datasets used and their main characteristics.

Dataset	Number of Identities	Number of Images	Image Dimensions	Training/Testing Partition Available	Pose Facilitation	Cloth Variation
CUHK03 [6]	1467	13,164	Varying	Yes	No	No
RAiD [7]	41	6920	128 × 64	Yes	No	No
Market-1501 [8]	1501	32,668	128 × 64	Yes	No	No
RGBD-ID [9,10]	11	22,845	640 × 480	Yes	No	Yes
Unified	2939	17,634	299 × 299	Yes	No	Yes
SOMAset [3]	50	100,000	200 × 400	No	Yes	Yes

**Table 2:** Accuracy at different ranks, per dataset, as achieved by the Inception V3 network.

Dataset	Rank-1	Rank-5	Rank-10
CUHK03	92.50%	98.31%	99.65%
RAiD	99.86%	100.0%	100.0%
Market-1501	82.11%	97.73%	99.58%
RGBD-ID	54.62%	59.46%	65.68%
Unified	54.28%	59.16%	65.42%

**Table 3:** Accuracy of the Inception V3 network at different ranks (Ranks-1, 5, 10), against state-of-the-art Re-Identification (Re-ID) methods, per dataset, for the identification scenario.

CUHK03 **Dataset**			
**Methodology**	**Rank-1 Rate**	**Rank-5 Rate**	**Rank-10 Rate**
[22]	**97.80%**	**99.60%**	**99.80%**
**Inception V3**	92.50%	98.31%	99.65%
[11]	87.50%	97.70%	98.90%
[3]	83.60%	97.50%	99.20%
[12]	82.10%	96.20%	98.20%
[16]	81.80%	95.20%	97.20%
[17]	79.40%	N/A	N/A
[23]	78.90%	N/A	N/A
[34]	77.90%	N/A	N/A
[18]	74.90%	92.90%	96.70%
[20]	74.68%	95.99%	97.47%
[19]	74.21%	94.33%	97.54%
[15]	72.30%	N/A	N/A
[26]	63.70%	80.60%	86.90%
[53]	62.70%	79.90%	86.20%
[28]	62.55%	90.05%	94.80%
[24]	61.36%	N/A	N/A
[25]	58.00%	N/A	N/A
[31]	52.20%	N/A	N/A
[27]	38.10%	N/A	N/A
[8]	24.30%	N/A	N/A
RAiD **Dataset**			
**Methodology**	**Rank-1 Rate**	**Rank-5 Rate**	**Rank-10 Rate**
**Inception V3**	**99.86%**	**100.0%**	**100.0%**
[3]	95.00%	100.0%	100.0%
[33]	59.84%	N/A	N/A
Market **-1501** **Dataset**			
**Methodology**	**Rank-1 Rate**	**Rank-5 Rate**	**Rank-10 Rate**
[30]	**98.10%**	**99.30%**	**99.60%**
[32]	96.20%	N/A	N/A
[23]	95.70%	98.40%	99.00%
[36]	95.40%	N/A	N/A
[34]	95.40%	N/A	N/A
[17]	95.30%	N/A	N/A
[35]	94.80%	N/A	N/A
[15]	94.80%	N/A	N/A
[22]	94.40%	N/A	N/A
[26]	93.80%	97.50%	98.50%
[53]	92.80%	97.30%	98.10%
[24]	92.13%	N/A	N/A
[25]	90.40%	N/A	N/A
[11]	88.90%	95.60%	N/A
[13]	87.04%	95.10%	96.42%
[21]	86.54%	95.16%	97.03%
[18]	83.31%	N/A	N/A
[16]	82.30%	92.30%	95.20%
**Inception V3**	82.11%	97.73%	**99.60%**
[19]	80.31%	N/A	N/A
[14]	79.51%	N/A	N/A
[3]	77.49%	91.81%	94.69%
[27]	77.11%	N/A	N/A
[49]	72.54%	N/A	N/A
[28]	61.02%	N/A	N/A
[8]	47.25%	N/A	N/A
RGBD-ID**Dataset**			
**Methodology**	**Rank-1 Rate**	**Rank-5 Rate**	**Rank-10 Rate**
[10]	**98.40%**	N/A	N/A
[38]	91.60%	N/A	N/A
[9]	85.20%	N/A	N/A
**Inception V3**	54.62%	**59.46%**	**65.68%**
[54]	30.10%	N/A	N/A
Unified **DATASET**			
**Methodology**	**Rank-1 Rate**	**Rank-5 Rate**	**Rank-10 Rate**
**Inception V3**	**54.28%**	**59.16%**	**65.42%**

**Table 4:** SOMAset accuracy at different ranks under cloth variation as achieved by the Inception V3 network.

Rank-1	Rank-5	Rank-10
15.70%	45.43%	66.84%

**Table 5:** SOMAset accuracy at different ranks under pose variation as achieved by the Inception V3 network.

Training Pose	Rank-1	Rank-5	Rank-10
Frontal	61.00%	88.15%	93.28%
Back	60.00%	89.33%	96.02%
**Side**	**77.00%**	**100.0%**	**100.0%**

**Table 6:** Verification accuracy per dataset as achieved by the Siamese network.

Dataset	Verification Accuracy
CUHK03	93.50%
RAiD	99.32%
Market-1501	99.10%
RGBD-ID	99.95%
Unified	92.27%

**Table 7:** mAP results of the Siamese network compared to state-of-the-art, per dataset, for the verification scenario.

CUHK03 **Dataset**	
**Methodology**	mAP
**Siamese**	**89.72%**
[3]	86.79%
[16]	84.80%
[18]	78.80%
[23]	76.90%
[17]	76.70%
[34]	73.00%
[15]	67.80%
[53]	57.60%
[26]	57.50%
[25]	56.50%
[24]	55.78%
[27]	40.30%
[8]	22.70%
RAiD **Dataset**	
**Methodology**	mAP
**Siamese**	**99.18%**
[3]	95.00%
Market **-1501 Dataset**	
**Methodology**	mAP
**Siamese**	**97.87%**
[36]	94.20%
[34]	94.20%
[22]	90.70%
[32]	89.70%
[23]	88.20%
[30]	87.60%
[17]	86.70%
[35]	86.00%
[15]	84.90%
[26]	81.60%
[24]	79.01%
[53]	78.70%
[11]	76.70%
[25]	76.01%
[21]	68.97%
[13]	66.89%
[27]	63.63%
[16]	62.10%
[14]	59.87%
[18]	59.69%
[19]	57.53%
[3]	53.50%
[49]	40.54%
[28]	35.68%
[8]	19.47%
RGBD-ID**Dataset**	
**Methodology**	mAP
**Siamese**	**99.95%**
[10]	99.50%
[9]	98.23%
[54]	88.70%
Unified **Dataset**	
**Methodology**	mAP
**Siamese**	**86.30%**

**Table 8:** Proposed Siamese versus Inception V3 network results per somatotype dataset.

CUHK03 **Dataset**	
Methodology	mAP
Siamese Network	89.72%
**Inception V3 Network**	**90.00%**
RAiD **Dataset**	
Methodology	mAP
**Siamese Network**	**99.18%**
Inception V3 Network	96.53%
Market **-1501** **Dataset**	
Methodology	mAP
**Siamese Network**	**97.87%**
Inception V3 Network	81.39%
RGBD-ID**Dataset**	
Methodology	mAP
**Siamese Network**	**99.95%**
Inception V3 Network	53.37%
Unified **Dataset**	
Methodology	mAP
**Siamese Network**	**86.30%**
Inception V3 Network	50.94%

**Table 9:** SOMAset verification accuracy under clothing and pose variations as achieved by the Siamese network.

Experiment	Verification Accuracy
Clothing variation	49.02%
Back pose	46.62%
Front Pose	46.80%
Side Pose	50.12%

**Table 10:** Mean values and standard deviations of the similarity scores, produced by the Inception V3 and the Siamese networks among subjects belonging to the same somatotype and subjects wearing same clothes.

		Similarity Scores	
**Comparison**	**Network**	**Mean Value**	**Standard Deviation**
Same somatotype/Different Clothes	Inception V3	0.9897912	3.7876 × $10^{-6}$
Same somatotype/Different Clothes	Inception V3	0.9087757	1.0225 × $10^{-2}$
Same somatotype/Different Clothes	Inception V3	0.9999905	1.0120 × $10^{-7}$
Same somatotype/Different Clothes	Inception V3	0.9889345	1.5363 × $10^{-3}$

**Table 11:** Advantages and disadvantages of the two proposed schemes.

Scheme	Advantages	Disadvantages
Inception V3 Network	- High identification ranks for most cases	- Cloth variation sensitive
	- Fast identification	- Re-training required for use with different datasets
Siamese Network	- High verification accuracy	- Slow identification
	- Fast verification	
	- Re-training is not required	
	- Less sensitive to cloth variation	
	- Small training set required	
